# Shotgun Metagenomics Reveals Systemic Metabolic Pathways Promoting Organic Acid Production in Traditional Sunki Fermentation

**DOI:** 10.3390/microorganisms14102395

**Published:** 2026-10-20

**Authors:** Asahi Oshima, Sona Sekiguchi, Shogo Uchikawa, Naoya Fujita, Hana Yoshida, Sachika Yamada, Nobuyuki Fujita, Yu Shiwa, Akira Tsuji, Naoto Tanaka, Sanae Okada, Akifumi Nishida

**Affiliations:** 1 Department of Molecular Microbiology, Tokyo University of Agriculture, Tokyo 156-8502, Japan; 2 Kiso Town Institute of Regional Resources, Nagano 397-8588, Japan; 3 Department of Fermentation Science, Tokyo University of Agriculture, Tokyo 156-8502, Japan

**Keywords:** shotgun metagenomics, lactic acid bacteria, salt-free fermentation, fermented vegetables, metabolic pathway, succinic acid, γ-aminobutyric acid shunt, microbial community

## Abstract

Salt-free lacto-fermentation is gaining attention owing to the global demand for sodium reduction. Sunki, a traditional unsalted Japanese turnip pickle, provides a unique ecological model. In this study, we investigated the microbial balance and systemic metabolic pathways that promote organic acid accumulation using shotgun metagenomics. We revealed that a high-quality sunki cluster exhibiting an abundant accumulation of organic acids (lactic acid 8.58 ± 2.42 g/L, acetic acid 2.70 ± 0.76 g/L, succinic acid 1.50 ± 0.52 g/L) and low pH (3.91 ± 0.15) was characterized by the co-dominance of specific lactic acid bacteria along with the foundational species *Limosilactobacillus fermentum*. This co-dominance enables the synergistic operation of both the homofermentative and heterofermentative pathways, whose functional potentials positively correlated with lactic acid (r = 0.50) and acetic acid (r = 0.48) concentrations, respectively, thereby achieving both effective preservation and flavor complexity. Furthermore, we propose a dual-pathway model for succinic acid production involving the reductive tricarboxylic acid cycle from malate and the γ-aminobutyric acid (GABA) shunt. Succinic acid positively correlated with the functional potential of the GABA shunt enzyme (*puuE*, r = 0.57) and negatively correlated with that of glutamate dehydrogenase (*gdhA*, r = −0.51), suggesting a metabolic switch redirecting glutamate flux toward the GABA shunt. This mechanism highlights an advanced ecological adaptation for pH homeostasis, emphasizing that a balanced co-dominance structure determines the quality of salt-free fermented foods.

## 1. Introduction

Fermented vegetable products have been produced worldwide since ancient times to improve preservation and impart flavor. In traditional pickle production, high salt concentrations are used to suppress the growth of spoilage microorganisms. However, driven by health-conscious demands for sodium reduction and sustainable food processing, salt-free lacto-fermentation has gained attention [1,2,3,4,5]. In this process, fermentation is controlled solely by the rapid proliferation of lactic acid bacteria (LAB) and subsequent acidification. In particular, fermented foods made from cruciferous (*Brassica*) vegetables, such as turnips and cabbages, contain abundant LAB and bioactive compounds and are globally recognized as beneficial foods that improve the intestinal environment and metabolism [3]. Generally, a low-pH environment associated with the accumulation of lactic acid as a metabolic byproduct (autoacidification) acts as a severe stress factor for LAB. Specifically, a decrease in intracellular pH is known to inhibit glycolytic enzymes, thereby reducing biochemical energy production [6] and significantly affecting the growth, survival, and structure of cell membranes and macromolecules [7,8]. Therefore, for the successful completion of the salt-free fermentation process, advanced acid adaptation mechanisms of the dominant LAB are crucial. Representative salt-free lactic acid-fermented foods include gundruk and sinki from the Himalayan region [9,10,11,12,13], and sunki from the Kiso region of Nagano Prefecture in Japan [2].

Sunki is a unique pickle made by fermenting the stems and leaves of red turnips without salt, the production of which has been passed down along with region-specific traditional techniques and the practice of subculturing starters [14]. Previous studies using cultivation methods, polymerase chain reaction–denaturing gradient gel electrophoresis [15], and 16S rRNA gene amplicon sequencing [16] have reported that LAB such as *Limosilactobacillus fermentum*, *Lactobacillus delbrueckii*, and *Lactiplantibacillus plantarum* dominate sunki fermentation. Furthermore, metabolomic analysis has suggested that differences in bacterial community structure affect the profiles of metabolites, such as acetic and succinic acids [17]. Importantly, the quality of sunki is determined by the abundant accumulation of organic acids such as lactic, acetic, and succinic acids, which drive the optimal pH decline and the formation of its distinctive sourness and flavor profile [16,17]. In other spontaneously fermented vegetables, such as shibazuke (a traditional Japanese fermented eggplant pickle), gundruk, and sinki, the combination of 16S rRNA gene analysis and functional prediction using PICRUSt2 [18] predicted that specific LAB contribute to carbohydrate metabolism and the conversion of amino acids (e.g., to glutamate) [19,20,21].

Microbial communities in fermented foods have become highly regarded as experimentally tractable microbial ecosystems for understanding the mechanisms of community formation in complex natural environments [22]. However, it has not been fully elucidated how the community systemically adapts its metabolism to maintain pH homeostasis under severe low-pH stress, and how specific community structures drive the abundant accumulation of organic acids (lactic, acetic, and succinic acids), which is essential for high-quality sunki. To address this question, we evaluated the functional genetic potential of the microbiota using shotgun metagenomic sequencing and a data-driven correlation approach on a wide range of samples submitted to the Sunki Competition. The primary objective of this study was to investigate the system-level metabolic networks driving the abundant accumulation of organic acids in a salt-free environment. Specifically, we aimed to identify the characteristic community structure and explore how the adaption to severe low-pH stress promotes the organic acid profile. Based on our findings, we propose an integrated biological adaptation model in which an adaptive metabolic route, the GABA shunt, serves dual roles in microbial acid tolerance and food flavor formation.

## 2. Materials and Methods

### 2.1. Sample Collection and Physicochemical Analysis

Sunki is traditionally produced by briefly parboiling the leaves and stems of red turnips in hot water to soften the plant tissues, followed by inoculation with a starter from the previous year (Figure S1). The analysis targeted sunki samples produced by individual households and submitted to the Sunki Competition, held annually in early December in Kiso Town, Nagano Prefecture, Japan. The samples were collected in 2017 (64 samples), 2018 (75 samples), 2022 (44 samples), 2023 (38 samples), 2024 (24 samples), and 2025 (39 samples). At the venue, pH was measured using a benchtop pH meter (SevenCompact pH/Ion S220; Mettler Toledo, Greifensee, Switzerland). Approximately 10 mL of the fermented juice was transported to the laboratory, and the organic acid concentration was measured using HPLC. A Chromaster Organic Acid (BTB) Analysis System (CH5110; Hitachi, Tokyo, Japan) utilizing the post-column method was used for organic acid analysis. The analytical conditions included a GL-C610H-S column (7.8 mm I.D. × 300 mm) and a GL-G-C600 guard column (4 mm I.D. × 10 mm), with the column temperature set to 60 °C. A perchloric acid solution was used as the eluent at a flow rate of 0.500 mL/min, and bromothymol blue solution was added as the reaction solution at 0.600 mL/min. Purified water was used as the autosampler wash solution and the detection wavelength was 440 nm. For samples collected between 2023 and 2025, microbial genomes were extracted and subjected to metagenomic analysis and organic acid measurements.

### 2.2. DNA Extraction and Metagenomic Sequencing

A 5 mL aliquot of fermented juice from 2023 to 2025 was centrifuged at 15,000× *g* for 5 min at 4 °C, and the pellet was collected after removing the supernatant. DNA was extracted using a DNeasy PowerSoil Pro Kit (QIAGEN, Hilden, Germany) according to the manufacturer’s protocol. The DNA concentration was measured using a 1× dsDNA HS Assay Kit and a Qubit 3 Fluorometer (Thermo Fisher Scientific, Waltham, MA, USA). Sequencing libraries were prepared using the NEBNext Ultra II FS DNA Library Prep Kit from Illumina (New England Biolabs, Ipswich, MA, USA), and library concentration and fragment length were confirmed using a TapeStation 4200 (Agilent Technologies, Santa Clara, CA, USA). Paired-end sequencing (2 × 150 bp) was performed using a MiniSeq next-generation sequencer (Illumina, San Diego, CA, USA) and a MiniSeq Mid Output Kit (300-cycles) (Illumina, San Diego, CA, USA).

### 2.3. Metagenomic Data Quality Control and Community Structure Analysis

The total number of reads obtained by sequencing is listed in Table S1. For quality control, fastp (v1.0.1) [23] was used with default settings for adapter removal and trimming of low-quality reads, and the data quality was checked using FastQC (v0.12.1) [24]. Samples with fewer than 50,000 reads after quality control (one from 2023 and two from 2025) were excluded from the subsequent metagenomic analyses. Community structure analysis was performed using Kraken2 (v2.17.1) [25] with the Standard database (14 July 2025, which consists of bacteria, archaea, viruses, and the human genome) with a confidence score of 0.1. Bracken (v3.0.1) [26] was then used with a read length of 150 bp and a classification level set for species to re-estimate relative abundances at the species level. Based on the microbiota profile of each sample, hierarchical clustering (unweighted pair group method with arithmetic mean) based on Bray–Curtis dissimilarity was performed using the major bacterial species with an average relative abundance of 1% or more. Dendrograms and heat maps were generated using the Seaborne Library [27] in Python (v3.11.14). To correct for variations in sequencing depth, species-level read count data were rarefied to a minimum library size (59,985 reads). Based on the rarefied count matrix, the Shannon diversity index and number of observed species were calculated. Differences in α-diversity among different metabolic clusters and across different years were evaluated using the Kruskal–Wallis test. For indicators showing significant overall differences, pairwise comparisons were conducted using the Mann–Whitney U test, and *p*-values were adjusted using the Benjamini–Hochberg procedure to control the false discovery rate (FDR).

### 2.4. Metagenome Assembly and Functional Gene Catalog Construction

For functional gene analysis, quality-controlled reads were assembled using metaSPAdes implemented in SPAdes (v4.2.0) [28]. Gene regions were predicted for the obtained contig sequences using Prodigal (v2.6.3) [29] in the metagenome mode (-p meta). The predicted nucleotide sequences were integrated and clustered using CD-HIT-EST (v4.8.1) [30] under the conditions of 95% sequence identity (-c 0.95), word size 10 (-n 10), and 90% alignment coverage for shorter sequences (-aS 0.9) to construct a non-redundant representative sequence catalog. Based on the ID lists of these representative nucleotide sequences, the corresponding amino acid sequences were extracted from the output of Prodigal to create an amino acid sequence catalog.

### 2.5. Functional Annotation and Metabolic Score Calculation

Functional annotation was assigned to the extracted amino acid sequence catalog using eggNOG-mapper (v2.1.13) [31] by performing homology searches against the eggNOG database (v5.0) [32] using the DIAMOND algorithm (-m diamond) [33]. Functional gene quantification was performed using Salmon (v1.10.3) [34]. After indexing the non-redundant nucleotide catalog reads from each sample were mapped using metagenome mode (--meta), automatic library type detection (-l A), and strict mapping validation (--validateMappings). Read counts were normalized to transcripts per million (TPM) and aggregated using KEGG Orthology (KO) identifiers [35] to create a functional profile matrix. To evaluate the functional potential of the Lactobacillales community, specific KO identifiers were selected as marker genes of key metabolic pathways. The homolactic fermentation potential was estimated using the sum of the abundance of fructose-bisphosphate aldolase genes (K01623 and K01624), as aldolase is the signature enzyme of the homofermentative Embden–Meyerhof pathway [1,36]. Heterolactic fermentation potential was evaluated using a specific phosphoketolase gene (K01621), which encodes the key branching enzyme that defines the heterofermentative phosphoketolase pathway [1,36,37]. In addition, the mean TPM of acetate kinase/phosphate acetyltransferase (K00925, K00625) [1,38] and fumarate reductase (K00244, K00245, K00246, and K00247) [1] were calculated as genetic potential scores for acetic and succinic acid production, respectively.

### 2.6. Correlation Analysis of Metabolic Profiles and Physicochemical Indicators

First, the correlation between predefined metabolic potential scores, measured organic acid concentrations (lactic, acetic, and succinic acids), and pH was evaluated. To reduce spurious correlations arising from the nature of the compositional data and eliminate fluctuations in absolute total amounts across years, a centered log-ratio (CLR) transformation was applied to both the metabolic potential and organic acid concentration datasets prior to correlation analysis. To handle zero values, pseudocounts of 0.1 and 0.01 were added to the metabolic potential data and organic acid concentration data, respectively, before performing CLR transformation using the vegan package [39] in the R environment (v4.3.3) [40]. Correlations between each metabolic potential and the organic acid profiles or physicochemical indicator (pH) were evaluated using Pearson correlation coefficient and *p*-values (significance level *p* < 0.05), and a correlation matrix was visualized using the corrplot (v0.95) package in R.

Next, to explore complex metabolic pathways (especially succinic acid production mechanisms) that could not be fully explained by predefined scores, a comprehensive correlation analysis (data-driven approach) between individual KOs and each organic acid concentration and pH was conducted. After extracting only the major KOs with an average TPM of one or more, pseudocounts (0.1 for KOs, 0.01 for organic acids) were added, followed by CLR transformation. Pearson correlation coefficients between each KO and target variables were calculated, *p*-values were adjusted using the Benjamini–Hochberg method, and an FDR < 0.05 was determined as a significant association. Functional annotations for each KO were assigned using the KEGG database [35] and KEGG REST API (accessed July 2026).

Additionally, Pearson correlation analysis was conducted between the relative abundances of the top 10 dominant bacterial species and the physicochemical parameters. Prior to the analysis, microbial abundances were CLR transformed (pseudocount 0.001) and pH was Z-score normalized. Significant correlations (*p* < 0.05) were visualized as a heatmap using the ggplot2 (v3.5.2) package in R.

### 2.7. Multivariate Analysis of Community Structure

Non-metric multidimensional scaling (NMDS) analysis was performed to visually and statistically evaluate differences in the community structure across years. For microbiota data (species-level abundance), rows and columns with all zeros were removed, Hellinger transformation was applied, and NMDS analysis based on Bray–Curtis dissimilarity was conducted. The overall community structure differences between years were tested using permutational multivariate analysis of variance (PERMANOVA) (adonis2 function) with 999 permutations, and *p*-values for post-hoc tests between groups (Pairwise PERMANOVA) were adjusted for multiple comparisons using the Benjamini–Hochberg method. The ggplot2 [41] and vegan packages in R were used to create NMDS ordination plots and draw 95% confidence ellipses, respectively.

### 2.8. Sample Categorization and Biomarker Discovery

To perform a comprehensive categorization of the samples based on the measured organic acids (lactic, acetic, and succinic acids) and pH data over the entire period (2017–2025), clustering was conducted using a Gaussian mixture model (GMM) with a full covariance matrix. For the organic acid concentration data, a pseudocount of 0.001 was added to prevent calculation errors, followed by CLR transformation, while the pH data were standardized (Z-score normalization). To determine the number of clusters (k), the Bayesian information criterion was calculated and evaluated in the range of *k* = 1–6 (Figure S2). Although the Bayesian information criterion score showed a minimum at *k* = 2, the 2-cluster classification resulted in similar competition award rates in each cluster, which was insufficient for extracting the characteristics of the awarded samples. Therefore, *k* = 3 was adopted as the final cluster number, because it clearly separated and explained the distribution and metabolic profile differences among the awarded samples. Principal component analysis (PCA) was performed to visualize the clustering results, generating a biplot of principal component scores and variable loadings. GMM clustering and PCA were performed using the scikit-learn package [42] in Python. Subsequently, the metabolic profile (mean ± standard deviation) and distribution of the awarded samples were evaluated for each cluster. Linear discriminant analysis effect size (LEfSe) was performed to identify the microbial species (biomarkers) characterizing these three clusters [43]. Species-level relative abundance data obtained using Bracken were multiplied by 1,000,000 to convert them into integer count equivalents, which were then combined with cluster information for each sample to create an input file. Subsequently, format conversion (lefse_format_input.py) was conducted using the LEfSe command-line tool with the GMM cluster information set as the comparison class. Then, linear discriminant analysis (lefse_run.py) was executed with a significance level of *p* < 0.05 (Kruskal–Wallis test), and a logarithmic linear discriminant analysis score threshold of 4.0. The effect size of each species was calculated, and the characteristic species were visualized (lefse_plot_res.py).

## 3. Results

### 3.1. Microbial Community Structure of Sunki

**Figure 1:**
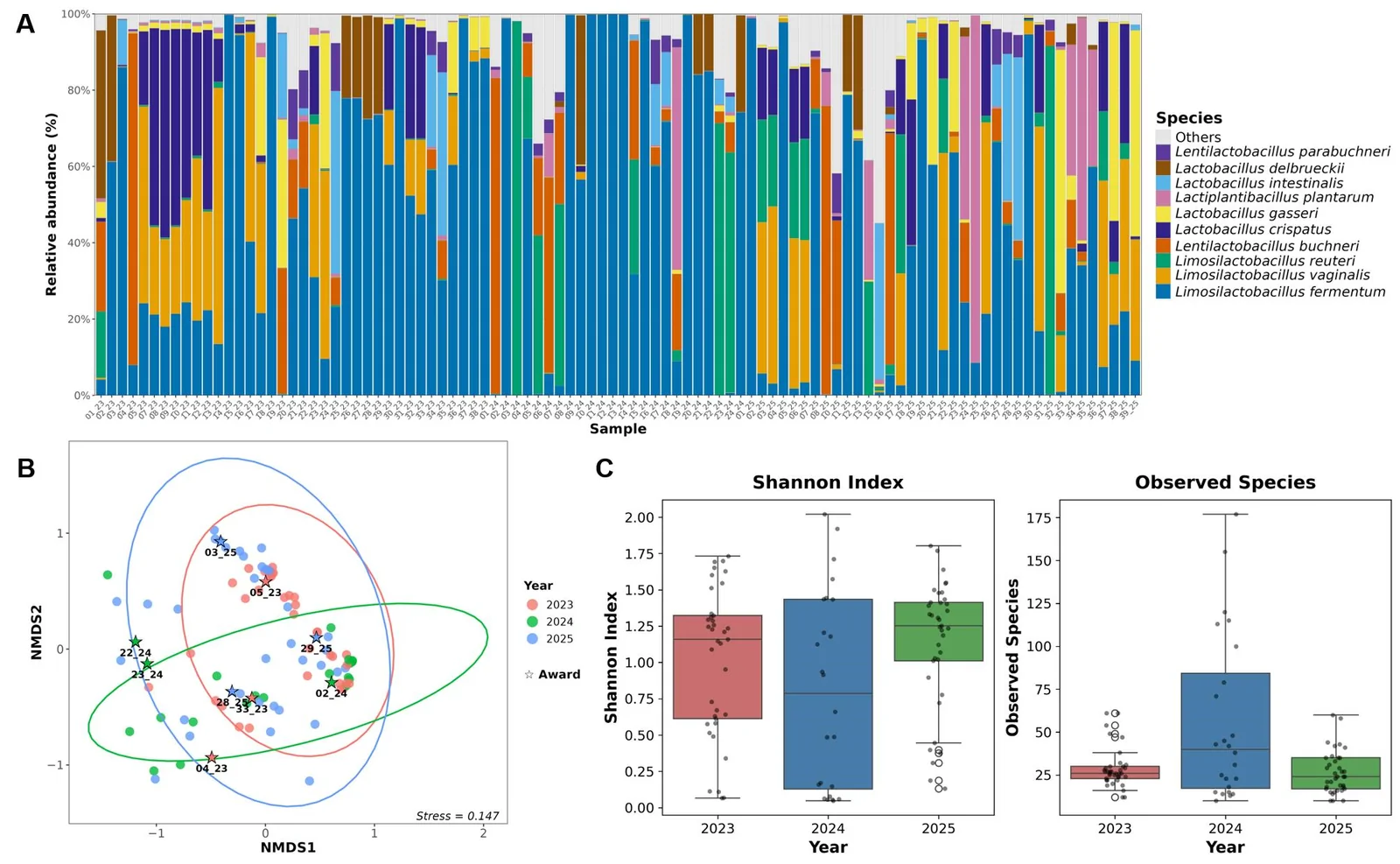
Microbial community structure of sunki fermented juice. (**A**) Relative abundances of the top 10 most abundant bacterial species across all samples are shown, with the remaining species aggregated into Others. (**B**) Non-metric multidimensional scaling (NMDS) plot of the microbial community structures across different years. Star symbols indicate the competition-awarded samples. (**C**) α-diversity of the microbiota (the Shannon index and the number of observed species) across different years. Statistical significance was evaluated using the Kruskal–Wallis test.

Taxonomic analysis (Kraken2/Bracken) of shotgun metagenomic sequencing data revealed the microbial community structure of sunki fermented juice at the species level. Overall, the microbiota was composed of 98.79% Lactobacillales, 0.37% other bacteria, 0.82% eukaryotes (entirely assigned to Homo sapiens, likely representing trace human DNA introduced during traditional household production and subsequent laboratory handling, as the employed database lacks fungal genomes), and 0.02% viruses. The most dominant bacterial species across all samples was *Limosilactobacillus fermentum*, which showed very high relative abundance (>99%) in many samples (Figure 1A). In addition, *Lactobacillus crispatus*, *Limosilactobacillus vaginalis*, *Lactobacillus delbrueckii*, *Lentilactobacillus buchneri*, and *Lactobacillus johnsonii* were frequently detected as major constituent species in high abundance. Hierarchical clustering based on Bray–Curtis dissimilarity using major species with an average relative abundance of 1% or more revealed that the sunki microbiota can be broadly classified into three types (Figure S3). The first group was a microbiota type in which *L. fermentum* was almost exclusively dominant with a very high abundance ratio. The second group was based on *L. fermentum* but was characterized by a high coexistence of *L. vaginalis* and *L. crispatus* as the major constituents. The third group exhibited a more diverse species composition, with other LAB such as *L. plantarum*, *L. buchneri*, *Lactobacillus intestinalis*, *Limosilactobacillus reuteri*, and *Lactobacillus gasseri* accounting for a major proportion. These results indicate that while *L. fermentum* functions as a foundational species in sunki fermentation, diverse and characteristic microbiota patterns are formed because of differences in the production environments, conditions, and starter cultures in each household. By comparing the sample metadata with the community structures, we found that within each of these sample groups (2023 samples 5 and 7–12, 26–28, 29–32, 35–38, and 2024 samples 10–13, 16–17, and 20–21), the sunki was produced using the same starter in the same household or in the elementary school, where sunki making is practiced as part of a local cultural education program. Consequently, even when raw turnips differed within the same group, the final microbiota type was consistent among the samples using the same starter. Furthermore, consistency in the dominant LAB species was observed between samples produced using the same 2023 sunki as a starter for the following year. Specifically, *L. buchneri* was dominant in 2023 sample 4 and 2024 sample 14; *L. fermentum* and *L. intestinalis* in 2023 sample 34 and 2024 sample 17; and *L. fermentum* and *L. delbrueckii* in 2023 sample 25 and 2024 sample 24, demonstrating that specific microbiota types were stably inherited across years.

### 3.2. Characteristics of Competition-Awarded Samples Based on Microbiota Structure and Organic Acid Composition

To evaluate the overall differences in the microbial community structure across the years, NMDS analysis was performed (Figure 1B). PERMANOVA testing revealed significant differences in the microbiota structure between years (Pairwise PERMANOVA with Benjamini–Hochberg adjustment: *p* < 0.05 for all pairwise comparisons). In terms of the α-diversity across different years (Figure 1C), while the Kruskal–Wallis test showed no significant overall differences in the Shannon index (*p* = 0.10) or the number of observed species (*p* = 0.12), the 2024 samples exhibited remarkably high variance, with several samples showing a substantially high number of observed species. When competition-awarded samples were mapped onto the two-dimensional NMDS space, they were widely dispersed across the entire plot without forming a cluster in a specific region. This indicates that the quality of sunki is not solely determined by a specific microbial community structure. To identify the robust metabolic characteristics that consistently define high-quality sunki regardless of year-to-year variations, samples from 2017 to 2025 were clustered using a GMM based on organic acid concentrations (succinic, lactic, and acetic acids) and pH.

**Figure 2:**
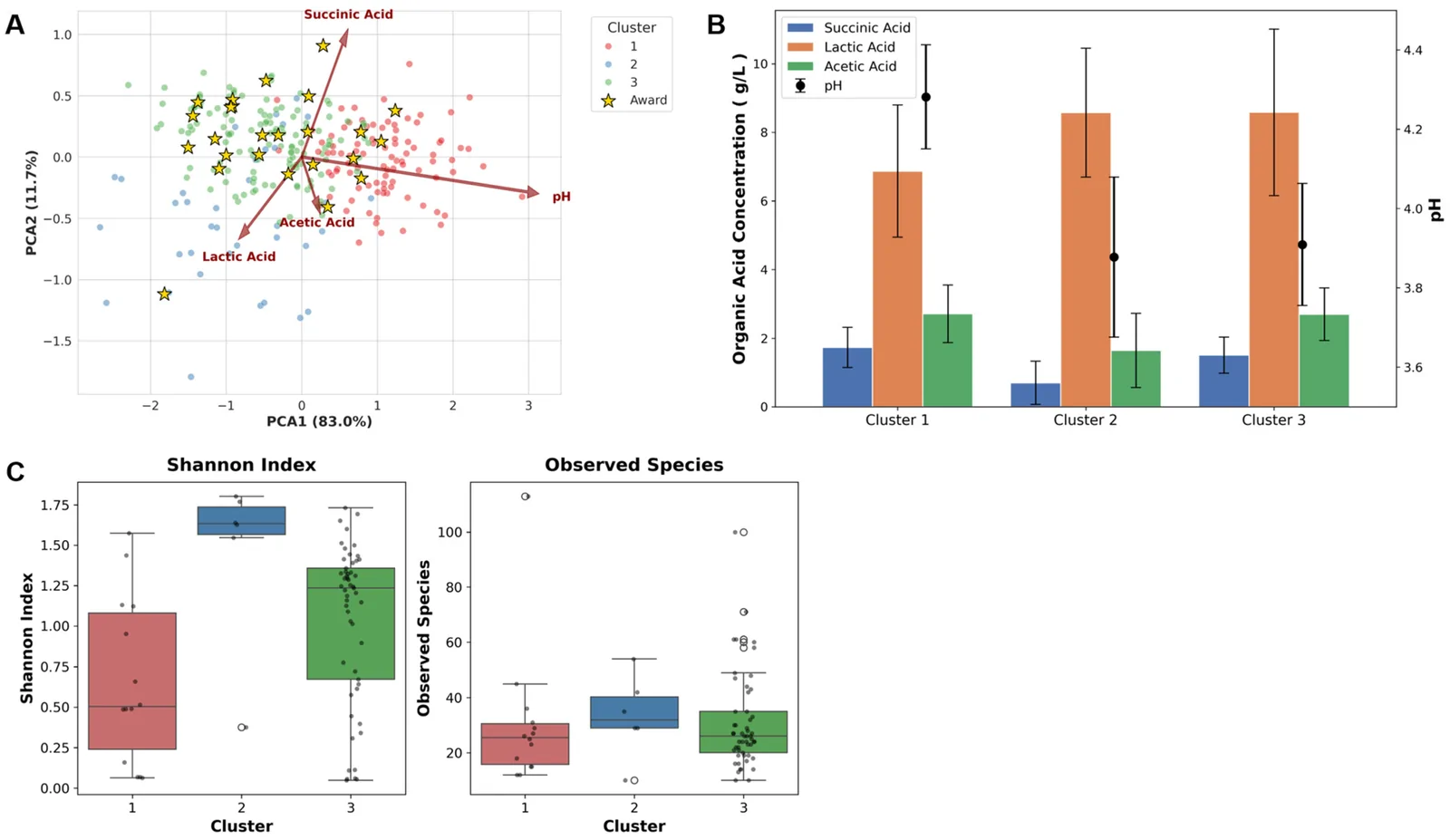
Characteristics of the three metabolic clusters categorized by the Gaussian mixture model (GMM). (**A**) Principal component analysis (PCA) biplot based on organic acid concentrations and pH. The arrows indicate the loading vectors for each variable. Star symbols indicate the competition-awarded samples. (**B**) Metabolic profiles (lactic, acetic, and succinic acid concentrations, and pH) of each cluster. Data are presented as mean ± standard deviation. (**C**) α-diversity of the microbiota (the Shannon index and the number of observed species) for each cluster. Statistical significance was evaluated using the Kruskal–Wallis test.

The samples were classified into three clusters (*k* = 3): clusters 1 (*n* = 102), 2 (*n* = 38), and 3 (*n* = 144) (Figure 2A). Cluster 1 showed a relatively high accumulation of succinic (1.73 ± 0.59 g/L) and acetic (2.71 ± 0.84 g/L) acids, but low lactic acid concentration (6.87 ± 1.93 g/L), resulting in a limited pH drop (pH 4.28 ± 0.13). Cluster 2 exhibited low concentration of succinic (0.70 ± 0.63 g/L) and acetic (1.64 ± 1.08 g/L) acids, but high in lactic acid (8.58 ± 1.88 g/L) and had a low pH (pH 3.88 ± 0.20). On the other hand, cluster 3 exhibited a comprehensive profile with abundant accumulation of all organic acids: succinic acid (1.50 ± 0.52 g/L), lactic acid (8.58 ± 2.42 g/L), and acetic acid (2.70 ± 0.76 g/L), along with a sufficiently lowered pH (pH 3.91 ± 0.15) (Figure 2B). Notably, a comparison of the distribution of awarded samples among the three clusters reveals a potential trend. The award rates were 6.9% (7/102), 5.3% (2/38) and 11.1% (16/144) in clusters 1, 2, and 3, respectively. Although not statistically significant, it is noteworthy that the majority of the awarded samples (16 out of 25, 64.0%) belonged to cluster 3, which was characterized by the abundant accumulation of organic acids.

Furthermore, to understand the community structure underlying these three clusters, the α-diversity of the microbiota was evaluated (Figure 2C). Interestingly, although no significant differences were found in the number of observed species between clusters (Kruskal–Wallis test, *p* = 0.506), the Shannon index, which reflects both species richness and evenness, showed a significant overall difference (*p* = 0.003). Post-hoc pairwise comparisons with FDR correction revealed that the Shannon index differed significantly among the three clusters (cluster 1 vs. 2: q = 0.013; cluster 1 vs. 3: q = 0.023; cluster 2 vs. 3: q = 0.013). This indicates that the microbial diversity features characterizing each metabolic cluster are driven primarily by differences in community evenness, rather than by the number of observed species.

### 3.3. Estimation of Characteristic Bacteria (Biomarkers) for Each Metabolic Cluster via LEfSe Analysis

**Figure 3:**
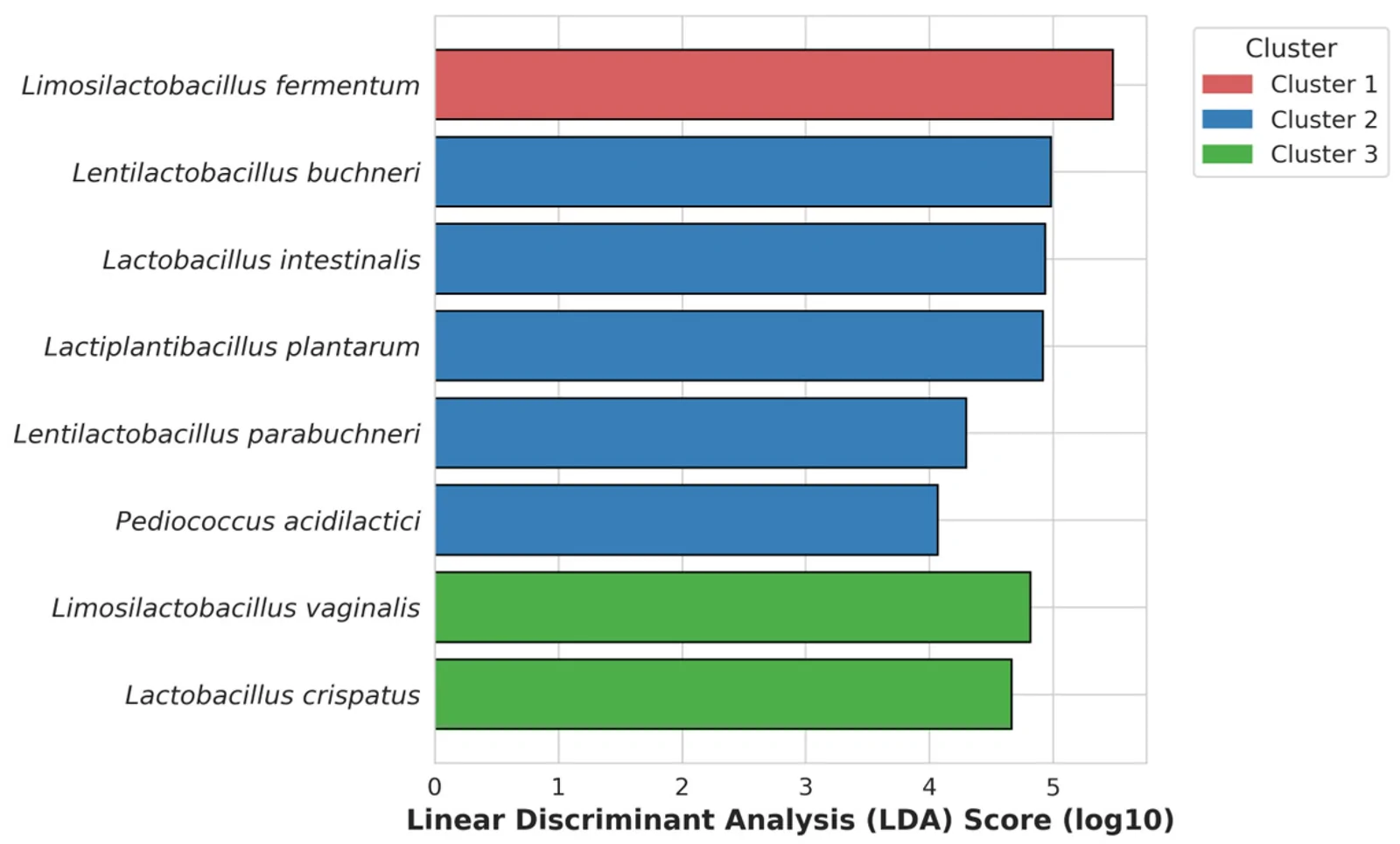
Identification of characteristic bacterial biomarkers for each metabolic cluster using linear discriminant analysis effect size (LEfSe) analysis. A logarithmic linear discriminant analysis (LDA) score threshold of 4.0 was applied.

To identify the specific microbial taxa characterizing each metabolic cluster, LEfSe analysis was performed (Figure 3). In cluster 1, characterized by high pH and low lactic acid concentration, *L. fermentum* was identified as the sole biomarker. In contrast, in cluster 2, which exhibited high lactic acid but low acetic and succinic acids, various LAB species, such as *L. buchneri*, *L. intestinalis*, *L. plantarum*, and *Lentilactobacillus parabuchneri* were identified as biomarkers. For cluster 3, which had the highest award rate and achieved abundant organic acid accumulation and a low pH, two species, *L. vaginalis* and *L. crispatus*, were identified as biomarkers. These results indicate that it is not the solitary dominance of *L. fermentum* (cluster 1) or variable species dominance (cluster 2) but a specific community pattern where *L. fermentum* serves as a foundational species with the co-dominance of *L. vaginalis* and *L. crispatus* (cluster 3) that is closely associated with abundant organic acid accumulation and the production of high-quality sunki.

### 3.4. Estimation of the Organic Acid Metabolic Pathways via Comprehensive Correlation Analysis

**Figure 4:**
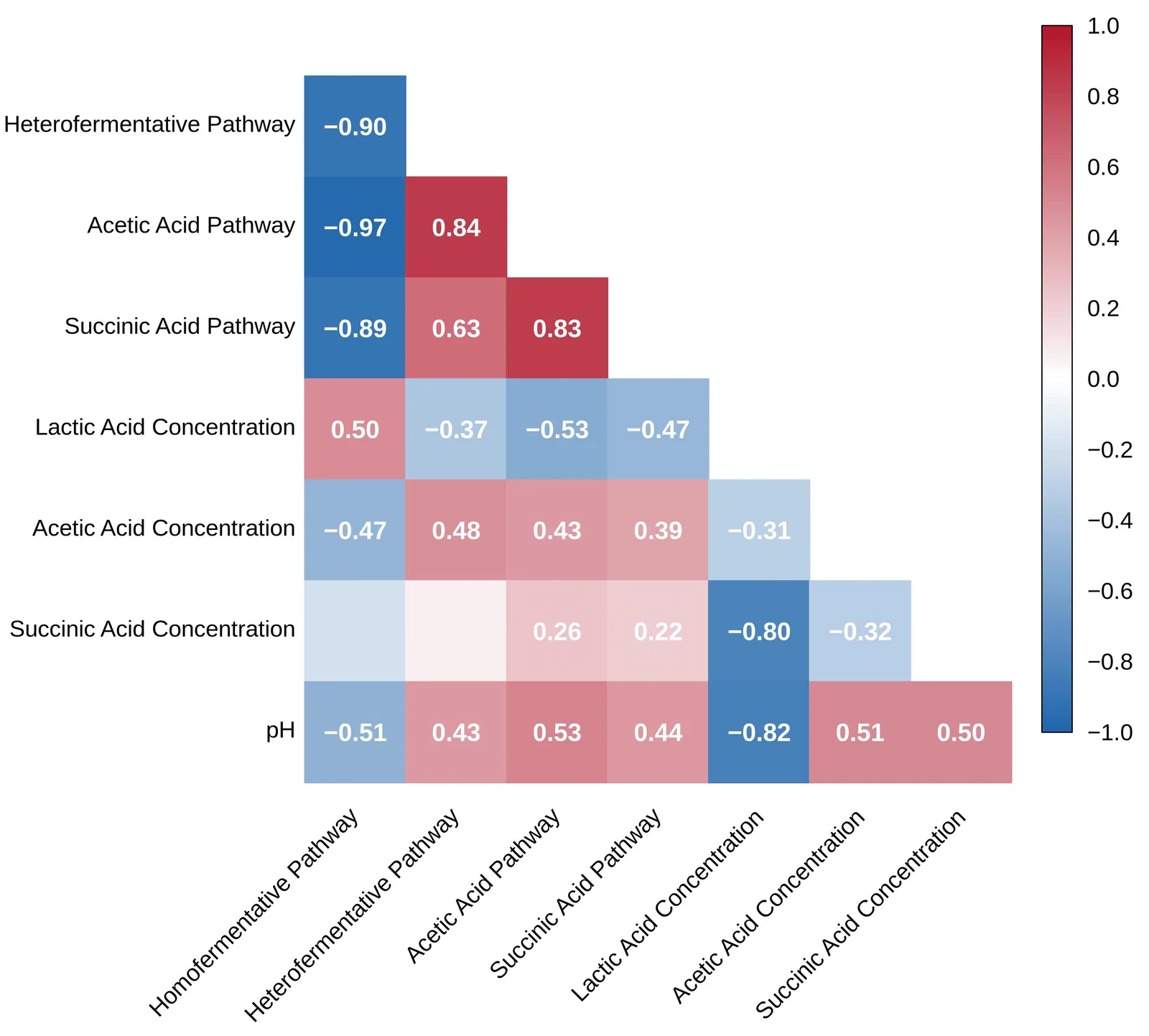
Correlation matrix between predefined metabolic potential scores, organic acid concentrations, and pH. The metabolic scores were calculated based on the transcripts per million (TPM) of specific functional marker genes. Only statistically significant Pearson correlation coefficients (*p* < 0.05) are displayed as numerical values within the cells.

Correlation analysis was performed between the metabolic markers calculated based on prior knowledge (homolactic fermentation, heterolactic fermentation, acetic acid synthesis, and succinic acid synthesis scores), organic acid concentrations, and pH (Figure 4). As a result, the homolactic fermentation potential showed strong negative correlations with the heterolactic fermentation potential (r = −0.90), the acetic acid production potential (r = −0.97), and the succinic acid production potential (r = −0.89). In contrast, the heterolactic fermentation potential positively correlated with the potential for acetic acid production (r = 0.84) and succinic acid production (r = 0.63). Regarding the actual metabolite concentrations, the homolactic fermentation potential was positively correlated with lactic acid concentration (r = 0.50), whereas the heterolactic fermentation potential was positively correlated with acetic acid concentration (r = 0.48). Furthermore, pH was negatively correlated with lactic acid concentration (r = −0.82) but positively correlated with acetic and succinic acid concentrations (r = 0.51 and r = 0.50, respectively). To identify the specific functional genes underlying these metabolic pathways, a comprehensive correlation analysis was conducted for all functionally annotated KOs. The results revealed the characteristic functional potential linkages for each organic acid (Data S3). Among the items showing significant correlations (FDR < 0.05), genes related to the phosphotransferase system sugar transporters for cellobiose and mannose (*celA*, r = 0.60; *manY*, r = 0.59), and genes related to cell envelope modification and peptidoglycan metabolism (*wecA* homolog involved in teichoic acid biosynthesis, r = 0.58; *murQ*, r = 0.58) were positively correlated with lactic acid concentration. In contrast, for acetic acid concentration, genes involved in amino acid metabolism, such as those involved in histidine biosynthesis (*hisD*, r = 0.53; *hisI*, r = 0.51), the shikimate pathway (*aroA*, r = 0.52; *aroK*, r = 0.51), and butanediol dehydrogenase (*butB*, r = 0.52) showed positive correlations, suggesting a shift to secondary fermentation pathways and secondary metabolism. Regarding succinic acid concentration, in addition to positive trends or correlations with fumarate hydratase (*fumA/B/C*, r = 0.18–0.38) and fumarate reductase (*frdA*, r = 0.37), which are involved in the conversion of malate to succinate via fumarate, positive correlations were observed with 4-aminobutyrate aminotransferase (*puuE*, r = 0.57) and succinate-semialdehyde dehydrogenase (*gabD*, r = 0.33), which are involved in the GABA shunt. In contrast, glutamate dehydrogenase (*gdhA*), which converts glutamate to α-ketoglutarate for entry into the TCA cycle, showed a negative correlation (r = −0.51). These results suggest that succinic acid production in sunki is strongly linked to the enriched genetic potential of a dual pathway involving the reductive TCA cycle and the GABA shunt utilizing red turnip-derived malate and glutamate, respectively, and that metabolic flux shifts toward the GABA shunt, coinciding with a relative decrease in *gdhA* gene potential.

To further evaluate these functional associations at the taxonomic level, a correlation analysis between the abundances of the top 10 major microbial species and the physicochemical parameters was conducted (Figure S4). The analysis revealed that the most dominant species, *L. fermentum*, exhibited positive correlations with acetic acid (r = 0.47) and succinic acid (r = 0.29), but a negative correlation with lactic acid (r = −0.57) and a positive correlation with pH (r = 0.64). Conversely, other major LAB species, such as *L. buchneri*, *L. plantarum*, and *L. intestinalis* showed positive correlations with lactic acid concentration (r = 0.30–0.33) and negative correlations with succinic acid (r = −0.45 to −0.26).

## 4. Discussion

### 4.1. Systemic Metabolic Adaptation of the Sunki Microbiota and Organic Acid Profiles

While previous studies have suggested a correlation between sunki community types and specific metabolites (e.g., lactic acid, acetic acid, and succinic acid) [16], our shotgun metagenomic analysis extended this understanding to the functional gene level. Our data-driven comprehensive correlation analysis revealed that the abundant accumulation of organic acids is underpinned by a system-wide metabolic adaption of the microbial community rather than relying on a single dominant species. Specifically, lactic acid concentration was positively correlated with the potential abundance of phosphotransferase system transporters for plant-derived sugars (e.g., *celA* and *manY*) and genes related to cell wall modification and synthesis (e.g., *wecA* homologs and *murQ*). In sunki’s production process, red turnips undergo a brief parboiling step before being mixed with the starter. This thermal treatment likely facilitates the partial disruption of plant cell walls, releasing intracellular nutrients and making structural polysaccharides more accessible for microbial degradation into fermentable sugars, such as cellobiose and mannose, in the matrix. Although comparative genomic analyses have shown that the repertoire of the phosphotransferase system and acid tolerance mechanisms varies significantly among LAB species and strains [37], these results suggest that specific LAB groups that possess enhanced genetic potential for the uptake of diverse plant tissue-derived sugars [44] and robust mechanisms to maintain and repair their cell walls under acid stress caused by autoacidification selectively dominate the community, thereby promoting efficient lactic acid fermentation. Previous transcriptomic and proteomic analyses have demonstrated that LAB and related Gram-positive bacteria upregulate or maintain the expression of sugar metabolism-related proteins, including the phosphotransferase system, under acid stress to ensure the energy supply necessary for survival [45,46]. In a competitive environment lacking the preservative effects of salt, the efficient uptake of sugars released by plant cell wall disruption and subsequent active acidification are considered essential strategies for eliminating spoilage, pathogenic, and other competing microorganisms, thus establishing the dominance of LAB [4,47].

In contrast, acetic acid production was positively correlated with genes involved in amino acid biosynthesis pathways (e.g., *hisD*, *hisI*, *aroA*, *aroK*) and butanediol dehydrogenase (*butB*), an enzyme involved in secondary fermentation. This suggests that an increase in acetic acid is part of a coordinated metabolic shift in response to environmental stress. Multiple omics analyses have revealed that when LAB face low pH stress due to autoacidification, they suppress lactate dehydrogenase activity. Instead, they reroute pyruvate metabolism toward a combination of survival strategies: producing acetic acid, engaging in secondary fermentation (e.g., butanediol production) to prevent further acidification, and synthesizing amino and fatty acids to repair cell structures and maintain membrane fluidity [6,48,49]. Because the resulting decrease in intracellular pH directly inhibits enzyme activity [6], such system-level metabolic shifts in response to severe low-pH stress during the late stages of fermentation are essential for survival and are presumed to concurrently contribute to the formation of the complex flavor profile characteristic of sunki.

### 4.2. The Dual-Pathway Model and Metabolic Switch Promoting Succinic Acid Production

Regarding succinic acid, which significantly contributes to the quality and complex umami of sunki, previous studies have suggested the involvement of the reductive TCA cycle utilizing malic acid derived from red turnip leaves [16,17]. Although succinic acid production from citric acid or malic acid by LAB has long been reported [50], our findings propose a dual-pathway model suggesting that the GABA shunt utilizing another abundant plant-derived substrate, glutamic acid, may function as an alternative route. Through data-driven analysis, a positive correlation was found not only with enzymes of the reductive TCA cycle (*fumA/B/C*, *frdA*), but also with 4-aminobutyrate aminotransferase (*puuE*) and succinate-semialdehyde dehydrogenase (*gabD*), which constitute the GABA shunt. Although GABA concentrations were not measured in this study, a previous NMR-based metabolomic analysis confirmed the presence of GABA in sunki [17]. The study showed an inverse relationship between the profiles of glutamic acid and GABA in PCA, reflecting their microbial interconversion during fermentation. In recent years, LAB have attracted attention as safe cell factories for GABA production in food fermentation. From a microbial physiological perspective, it has been proposed that this GABA shunt not only converts GABA into succinic acid to supply the incomplete TCA cycle but also plays a crucial role in maintaining pH homeostasis (acid tolerance) through intracellular proton consumption in acidic environments [51,52,53], which strongly aligns with our proposed model.

Certain LAB strains convert extracellular glutamic acid into GABA through uptake and subsequent decarboxylation by glutamate decarboxylase. This system is an important acid tolerance mechanism that maintains pH homeostasis by consuming intracellular protons [7,54,55]. Similarly, in metagenomic analyses of gundruk, a comparable unsalted fermented vegetable, it was confirmed that the *gad* operon (*gadA*, *gadB*, *gadC*), which is involved in acid tolerance and GABA production, is highly conserved in the metagenome-assembled genomes of recovered LAB [10], suggesting that this is a common adaptive strategy for surviving harsh and low-pH environments. In addition, coupling the electrogenic antiport of glutamic acid and GABA with the decarboxylation reaction generates a proton motive force that contributes to cellular ATP synthesis [54]. Furthermore, a significant negative correlation was observed with the potential abundance of glutamate dehydrogenase (*gdhA*). The relative decrease in the *gdhA* gene in the metagenome suggests that this suppression functions as an ecological switch, restricting the flow of glutamic acid into the oxidative branch of the TCA cycle at the community level and redirecting the metabolic flux toward the GABA shunt. Gong et al. demonstrated that in *Levilactobacillus brevis* transcription factors (such as GlnR) precisely regulate the expression of related genes (e.g., *gadB* and *gadC*) to control the balance between the acid stress response and GABA conversion [56]. This regulatory mechanism highlights an advanced biochemical symbiotic relationship between microorganisms and plant substrates, in which succinic acid accumulation is closely associated with a pH homeostasis maintenance mechanism that actively consumes protons to acquire energy and protect cells under highly acidic conditions.

### 4.3. Ecological Convergence and the Role of Beneficial LAB Characterizing High-Quality Sunki

The highly adapted metabolic system in sunki is not achieved by a single bacterial species but is formed through the interaction of environmental factors and human-mediated selection. While previous study reported that specific LAB groups are stably maintained during the subculturing process in each household [15], our analysis further demonstrated that when the same starter was used, the microbiota types remained consistent even if the raw turnips differed, and the dominant bacterial species were stably inherited across years. In similar unsalted naturally fermented vegetables such as gundruk and sinki, it has also been confirmed that spoilage bacteria are eliminated as fermentation progresses, and the community shifts toward dominance by specific LAB, such as *L. plantarum* [9,57,58]. Generally, in naturally fermented pickles, the introduction of a starter and maintenance of an appropriate initial microbiota have been reported to rapidly suppress the growth of spoilage bacteria, promote the colonization of LAB, shorten the fermentation process, stabilize the quality, and produce specific metabolites (organic acids and umami amino acids) [19,59]. In a previous study, a community exclusively dominated by *L. delbrueckii* was reported in sunki samples from 2015 [16]; however, such a microbiota was not observed in the samples from our study (2023–2025). This transition in community structure reflects ecological convergence through cultural and ethnobotanical fermentation techniques, such as the sharing and subculturing of traditional starters in the Kiso region [14]. It is presumed that, as sunki with a desirable flavor continued to be selectively subcultured and shared among producers, minor microbial populations that caused quality variations were eliminated, optimizing the regional fermentation ecosystem toward a stable microbial community centered on the foundational species, *L. fermentum*. However, this highly optimized system remains susceptible to meteorological fluctuations. For instance, according to insights from the Sunki Competition, lactic acid fermentation stagnated in 2024 due to a several-week delay in the first frost. In *Brassica* vegetables like turnips, cold stress from frost is essential to trigger the accumulation of sugars as a cryoprotectant. This delayed cold exposure prevented the sugar content of the red turnips from increasing sufficiently, leaving the LAB with a shortage of fermentable carbohydrates. The resulting fermentation stagnation and insufficient lactic acid accumulation caused many households to abandon their competition entries and likely explain why several samples in 2024 exhibited a substantially high number of observed species. Such instances demonstrate that the initial sugar content, which depends on meteorological conditions, significantly affects fermentation efficiency and community structure, highlighting that environmental factors can override even the use of a robust starter.

Through shotgun metagenomic analysis, we identified *L. crispatus* and *L. vaginalis* at the species level. The latter had previously been subsumed into higher phylogenetic groups, such as the *L. reuteri* group in 16S rRNA analyses, owing to high sequence similarity [16]. The α-diversity analysis showed that high-quality sunki (cluster 3) avoided the solitary dominance of *L. fermentum* (cluster 1) and variable species dominance (cluster 2), and that these characteristic bacteria co-dominated while maintaining high evenness. *Lactobacillus crispatus* and *L. vaginalis* have been reported to contribute to the production of aroma compounds, such as diacetyl and acetoin, and exhibit high antimicrobial activity against food-spoilage bacteria and pathogens through the production of high concentrations of acetic and lactic acids [60]. Additionally, *L. vaginalis* has been shown to possess bifidogenic and anti-inflammatory properties [61]. Therefore, the coexistence of these beneficial bacteria is considered to play a crucial role not only in stably supporting complex metabolic pathways but also in enhancing the flavor, safety, and probiotic functionality of sunki.

Our metagenomic and metabolic analyses collectively highlight a crucial ecological and metabolic mechanism shaping sunki quality: the balanced coexistence of both homofermentative and heterofermentative pathways. While the homofermentative potential promotes the accumulation of lactic acid necessary for environmental acidification and preservation (r = 0.50), the heterofermentative potential is positively correlated with acetic acid concentration (r = 0.48), contributing to flavor complexity. Rather than relying on a single metabolic regime, high-quality sunki (cluster 3) achieved abundant accumulation of organic acids through the complementary operation of these diverse pathways within the co-dominant LAB community, ultimately characterizing the high quality of the awarded samples.

While this study provides comprehensive insights into the sunki microbiota and its metabolic pathways, certain limitations should be addressed in future research. First, although our study demonstrated that specific microbiota types are stably inherited when the same starter is used, we did not directly sequence the starters themselves. Therefore, the exact contribution of starter diversity to the overall variations in community structure across different years remains to be fully elucidated. Second, although our data-driven approach proposed a dual-pathway model for succinic acid production and a metabolic switch toward the GABA shunt, our analysis is based on the functional genetic potential rather than the active metabolic state, lacking direct measurement of actual gene expression or real-time metabolic fluxes, and other quality-related metabolites such as GABA and volatile flavor compounds. Therefore, to experimentally verify the functional dynamics of the proposed metabolic pathways under unsalted and low-pH conditions, time-series multiomics analyses integrating metatranscriptomics and metabolomics are indispensable for future research.

## 5. Conclusions

In this study, shotgun metagenomics and data-driven correlation analyses were used to elucidate the complex ecological and metabolic interactions that promote organic acid accumulation in high-quality sunki, a traditional salt-free fermented vegetable. We demonstrated that the quality and abundant accumulation of organic acids in sunki are not dictated by the solitary dominance of the foundational species *L. fermentum* but rather by an ecologically balanced co-dominance with specific beneficial LAB, such as *L. crispatus* and *L. vaginalis*. This co-dominant structure is crucial for supporting the complementary operation of both homofermentative and heterofermentative pathways, ensuring sufficient acidification for preservation, while imparting flavor complexity through acetic acid production. Furthermore, comprehensive functional profiling revealed a system-wide metabolic adaptation essential for survival in severe autoacidification and salt-free competitive environments. Crucially, we propose a dual-pathway model for succinic acid production, highlighting the concurrent potential of the reductive TCA cycle and the GABA shunt utilizing malate and glutamate, respectively. The relative decrease in glutamate dehydrogenase (*gdhA*) potential functions as an ecological switch, redirecting glutamate flux toward the GABA shunt. This rerouting not only facilitates succinic acid accumulation but also contributes to maintaining pH homeostasis through intracellular proton consumption, representing a sophisticated biochemical interplay between the microbial community and plant substrates. Integrating metatranscriptomic and metabolomic approaches is vital for future research to further validate the real-time dynamics of these proposed metabolic pathways. The systemic adaptation model of the microbiota proposed in this study underscores the critical role of microbial community evenness and ecological co-dominance in shaping the quality, safety, and flavor of salt-free fermented foods, thereby providing a fundamental basis for optimizing salt-free lacto-fermentation processes.

## Data Availability

The raw shotgun metagenomic sequencing data presented in this study are openly available in the NCBI Sequence Read Archive under BioProject accession number PRJNA1519966.

## Supplementary Materials

The following supporting information can be downloaded at https://www.mdpi.com/article/10.3390/microorganisms14102395/s1: Figure S1: Overview of the traditional production process of sunki; Figure S2: Evaluation of Bayesian information criterion (BIC) scores for determining the optimal number of clusters; Figure S3: Hierarchical clustering dendrogram based on Bray–Curtis dissimilarity of the Sunki microbiota; Figure S4: Correlation heatmap between the relative abundances of the top 10 dominant microbial species and physicochemical parameters; Table S1: Summary of metagenomic sequencing data and read processing statistics; Data S1: Species-level microbial read count data generated by Kraken2/Bracken; Data S2: Raw data of organic acid concentrations and pH, and competition results (Awarded: 1 = Yes, 0 = No) of Sunki samples from 2017 to 2025. Data S3: Comprehensive correlation analysis results between all functionally annotated KOs and organic acid concentrations.

## Abbreviations

The following abbreviations are used in this manuscript:

CLR	Centered log-ratio
FDR	False discovery rate
GABA	γ-aminobutyric acid
GMM	Gaussian mixture model
KO	KEGG orthology
LAB	Lactic acid bacteria
LefSe	Linear discriminant analysis effect size
NMDS	Non-metric multidimensional scaling
PCA	Principal component analysis
PERMANOVA	Permutational multivariate analysis of variance
TCA	Tricarboxylic acid cycle
